# High levels of maternally derived antibodies do not significantly interfere with the development of humoral and cell-mediated responses to *Porcine circovirus 2* after intradermal vaccination

**DOI:** 10.1186/s40813-023-00335-9

**Published:** 2023-09-15

**Authors:** Gerard E. Martin-Valls, Martí Cortey, Hanny Swam, Marta Jiménez, Enric Mateu

**Affiliations:** 1 Departament de Sanitat i Anatomia Animals, Universitat Autònoma de Barcelona, Travessera dels Turons s/n, 08193 Cerdanyola del Vallès, Spain; 2 MSD CDS, 5831 AN Boxmeer, The Netherlands; 3 MSD Animal Health, 37008 Carbajosa de la Sagrada, Spain

**Keywords:** Porcine circovirus 2, Maternally derived antibodies, Interference, Immune response

## Abstract

**Background:**

Vaccination of pigs against PCV2 is usually performed around weaning when animals still have maternally derived antibodies (MDA). The present study aimed to assess the possible interference of MDA in the development of the PCV2-specific immune response after vaccination of commercial weaners. For this purpose, a PRRS-negative 600-sow farrow-to-finish farm was selected. Half of the sows were vaccinated and revaccinated with Porcilis® PCV ID against PCV2 7 and 3 weeks before farrowing. After farrowing, piglets were tested by AlphaLisa to select 72 animals with high and low levels of MDA. Groups were further subdivided and vaccinated intradermally with Porcilis® PCV ID at 21 or 28 days of age. Unvaccinated controls were also included. Animals were followed afterward for 42 days to examine the development of PCV2-specific antibodies and interferon-γ secreting cells (IFN-γ SC).

**Results:**

The average titres of antibodies of the groups vaccinated in the presence of low or high MDA levels were similar at 28 and 42 days post-vaccination while in the controls the titres declined throughout the observation period. Results of vaccinating at 21 or 28 days of age were equivalent with regard to antibody development. Regarding the IFN-γ SC, vaccinated animals produced significant frequencies of IFN-γ SC by day 28. Again, no differences were observed between the groups with high or low antibody levels.

**Conclusion:**

High levels of MDA did not interfere with the development of humoral and cell-mediated responses to Porcine circovirus 2 after intradermal vaccination at 21 or 28 days of age.

**Supplementary Information:**

The online version contains supplementary material available at 10.1186/s40813-023-00335-9.

## Background

Porcine circovirus 2 (PCV2)-associated diseases emerged in the decade of 1990, becoming one of the most feared threats to the swine industry. At that time, the main manifestation of the infection in the herds was a wasting disease, affecting a variable proportion of weaners and growers that usually ended up with the death of the affected animals [1–3]. Since then the infection became endemic in the domestic pig population.

The pharmaceutical industry reacted to this emergency by developing vaccines that were proven to be very efficacious. As a result, PCV2 vaccination became part of routine immunization programs for piglets. In addition, vaccines for sows were also licensed. The vaccination of sows aimed to reduce lesions in lymphoid tissues associated with PCV2 infection and as an aid to reduce PCV2-associated mortality in piglets due to passive immunization via the colostrum. However, a reduction of vertical transmission to piglets [4], as well as protection against the reproductive effects of the infection [5] were also observed. Interestingly, when pre-mating vaccination of sows is implemented the higher MDA titers in colostrum may cause interference with the development of antibodies against PCV2 after vaccination of piglets [6].

In endemic farms, infection of piglets can happen by different routes [7]. Vertical transmission is a well-documented one [8, 9] but often, piglets are infected when maternally derived antibodies (MDA) fade out, around 6–8 weeks of age [10]. Accordingly, vaccination of piglets must be performed at earlier ages, to warrant enough time for developing an adequate immune response before the loss of MDA. However, the earlier the vaccination, the higher the levels of MDA and, consequently, the higher the potential for the MDA levels interfering with the development of PCV2-specific immune responses after vaccination. However, any impairment in the development of the immune response after vaccination does not necessarily equate to lack of protection, as long as sufficient immunity is induced to prevent the development of clinical signs and lesions. Both the development of neutralizing antibodies and the cell-mediated immunity have been considered correlates of protection [11–13] but a precise cut-off for the protection has not been established. The evidence is that, in general, when PCV2 vaccines are administered at weaning in endemic farms, they are effective, although administration of the vaccine to animals with high levels of MDA may result in lack of clear seroconversion [14]. In another study, Fraile et al. [15] established a negative correlation between the titers of MDA the day of vaccination and the increase of antibody titers 21 days after vaccination. One of the approaches to overcome MDA interference is to implement a double vaccination protocol, usually in a 3 or 4-week period. This approach is troublesome since it implies an increase in the medication and labour costs.

Alternative routes of vaccine administration showing less interference with the MDA could be a solution to overcome this problem. It has been suggested that intradermal vaccination could be useful to reduce the interference created by MDA. For example, in humans, it was shown that the intradermal vaccination of new-borns against poliovirus, using an inactivated vaccine at one-fifth of the intramuscular dose, produced an immunization equivalent to that of the full-dose intramuscular vaccine [16].

The present study aimed to elucidate whether the intradermal vaccination against PCV2 can overcome the interference produced by MDA at two different ages, 21 and 28 days of age with the development of the humoral and cell-mediated responses.

## Results

**Fig. 1:**
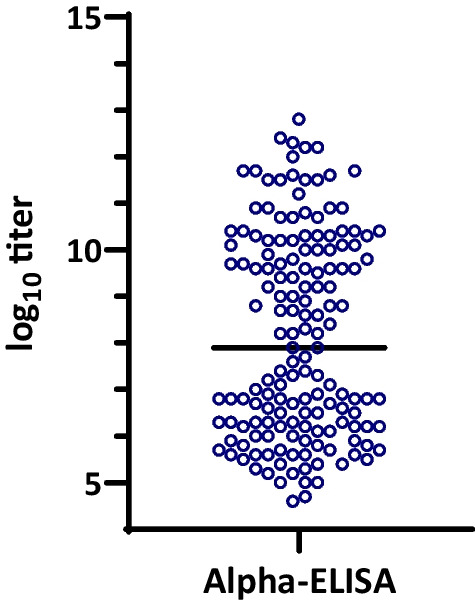
Distribution of anti-PCV2 antibody titres at 7 days of age using the PCV2 AlphaLISA. The graph shows the individual values for each serum and the median of the 161 piglets examined (horizontal bar). Animals in the high antibody level subgroup (offspring of vaccinated sows) were located above the median and animals in the low antibody subgroup (offspring of non-vaccinated sows) were located below the median

At 7 days of age, all animals had detectable levels of anti-PCV2 antibodies although the offspring of vaccinated sows had significantly higher levels (*p* < 0.05). Figure 1 shows the distribution of antibody levels at that age in the PCV2 AlphaLisa technique. Results obtained with the PCV2 ELISA (Biocheck®)and a comparison of results between both methods are shown in Additional file 1: Fig. S1 and Additional file 2: Fig. S2. At 7 days of age, all animals were negative for PCV2 by PCR (results not shown) and remained so for the rest of the study.

**Fig. 2:**
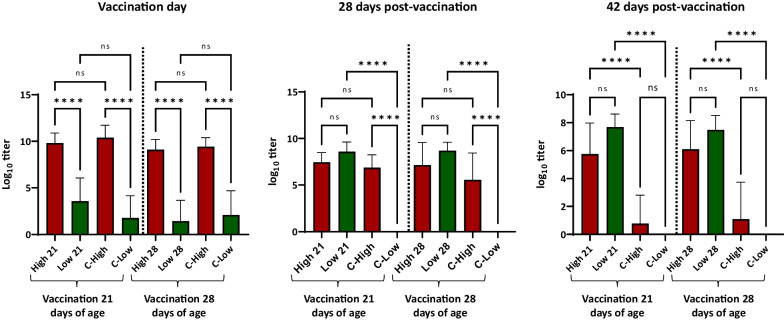
Average and standard deviation of the anti-PCV2 antibody titres as determined by AlphaLisa at different time points after vaccination. High 21, Low 21 = animals with high or low levels of antibodies vaccinated at 21 days of age; High 28, Low 28 = animals with low levels of anti-PCV2 antibodies vaccinated at 28 days of age, C-high = Controls selected with high levels of antibodies; C-Low: controls selected with low levels of antibodies. *p < 0.05; **p < 0.01; ***p < 0.001; ****p < 0.0001; *ns = *non-significant differences

Figure 2 summarizes the evolution of the anti-PCV2 antibodies in the different groups using the AlphaLisa. On the day of vaccination of each group, the serological analyses confirmed that the levels of anti-PCV2 antibodies for the high and low-level groups were different but were similar to their respective controls. At 28 days post-vaccination, all selected controls with low levels of antibodies were already seronegative. At that moment, the average titres of antibodies of the vaccinated groups were similar. At 42 days post-vaccination, all animals in the control groups were seronegative. In contrast, most animals in the vaccinated groups were seropositive. No statistically significant differences were found between the levels of antibodies in the animals vaccinated at 21 or 28 days of age in the presence of either high or low levels of MDA.

**Fig. 3:**
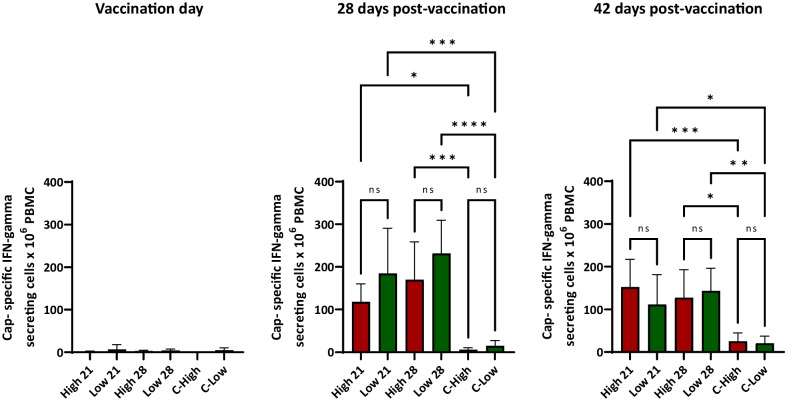
Levels of PCV2-specific IFN-γ secreting cells as determined by ELISPOT. Frequencies of PCV2-specific IFN-γ secreting cells at different timepoints after vaccination (mean and standard deviation). *p < 0.05; **p < 0.01; ***p < 0.001; ****p < 0.0001; *ns = *non-significant differences

The analysis of the IFN-γ responses by ELISPOT (Fig. 3) showed that piglets did not have any cellular immune response against PCV2 on the day of vaccination. Afterwards, the vaccinated animals developed a cell-mediated response as indicated by the rising frequencies of PCV2-specific IFN-γ-secreting cells (IFN-γ-SC). The only difference between groups was observed at 28 days post-vaccination when animals vaccinated at 21 days in the presence of high antibody levels had lower frequencies of IFN-γ-SC (*p* < 0.05) compared to the animals with low antibody levels vaccinated at 28 days of age. At 42 days post-vaccination, that difference was not observed. Frequencies of IFN-γ-SC of controls at 28 and 24 dpv were very low and non-significant compared to the results recorded for the controls groups at the beginning of the experiment (0.2 and 2.5 PCV2 IFN-γ producing cells per million PBMC on average for the C-High and C-low groups, respectively).

**Fig. 4:**
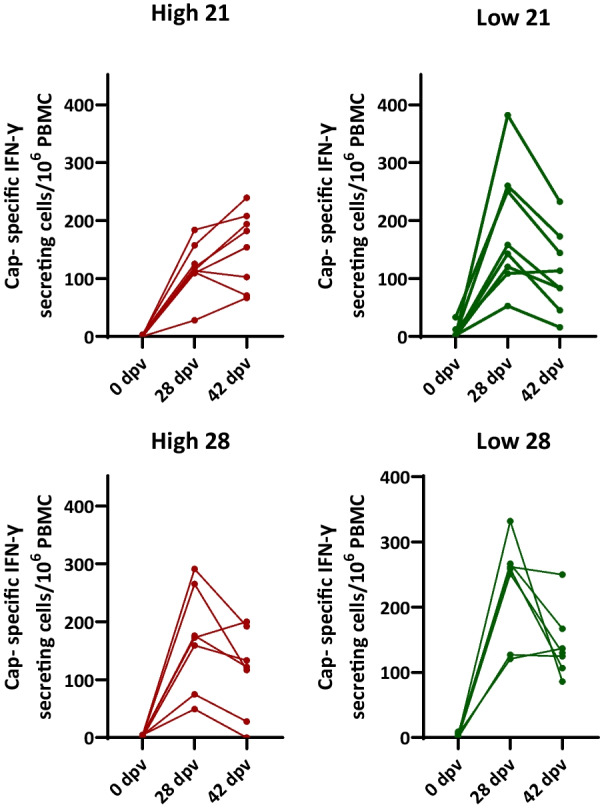
Individual responses of PCV2-specific IFN-γ secreting cells as determined by ELISPOT. The graphs show the frequencies of PCV2-specific IFN-γ producing cells only for the vaccinated groups

The evaluation of the individual PCV2-specific IFN-γ responses (Fig. 4) showed similar dynamics in all groups, regardless of the antibody titres or the day of vaccination. Of note, in all groups there were high and low responders. There was a trend for higher peak responses in the low antibody subgroup vaccinated at 21 days compared to the high antibody subgroup vaccinated that same day.

## Discussion

Vaccination of piglets is crucial to prevent the development of diseases that can have a serious impact on the herd. In general, vaccination strategies for piglets seek to develop active immunity in the animal before the time when a likely exposure to the pathogen may occur. Nevertheless, in the offspring of immune individuals, the earlier the vaccination time, the higher the levels of MDA. MDA are helpful to protect against the development of PCV2-associated diseases although they may not fully protect against infection [17]. However, if vaccination is delayed until the time when MDA are already undetectable, the risk of infection before the establishment of active immunity by vaccination increases. Interference with the development of immunity after vaccination by MDA is thought to affect mostly the development of humoral responses [18].

Vaccination against PCV2 has been proven to be highly effective to control PCV2-associated diseases [19–21]. Most PCV2 vaccines are intended for use in piglets older than 2–3 weeks of age, when piglets can still have MDA. Therefore, interference with MDA can be potentially significant. This could be particularly important when sows are also vaccinated against PCV2 to prevent PCV2-associated reproductive disease and to enhance the passive protection of the piglets. With regards to this, Sibila et al. [22] have shown that the highest levels of MDA in piglets were those of the offspring of sows vaccinated against PCV2. The present study examined this scenario of piglets with high MDA, in which sows were vaccinated before farrowing to increase the levels of MDA in piglets.

The distribution of anti-PCV2 titres before vaccination in the piglets of the present study allowed the identification of a group with high levels of antibodies, the offspring of vaccinated sows, and a group with low levels of antibodies, the offspring of unvaccinated sows. These results confirmed that vaccination of sows results in a significant increase of MDA in the piglets. It is worth noting that the two groups were well identified by the two ELISAs used, although the AlphaLisa allowed a better discrimination than the Biocheck ELISA. The observed evolution of the humoral response in the offspring of vaccinated sows strongly indicates that intradermal vaccination of animals with high MDA levels was not affected by a significant interference. Comparison of MDA levels among different studies is difficult because of the diversity of techniques used; however, in other studies where sows were vaccinated by the intramuscular route, interference was observed [6, 15] when titers were well above the average of the population. In other cases, vaccination in the presence of high titers of MDA had no practical impact. For example, Figueras-Gourgues et al. [23] examined data of more than 6000 pigs corresponding to four PCV2 vaccination field trials and concluded that MDA did not interfere with regards to the average daily weight gains or the clinical protection. Data from the present study showed that 42 days post-vaccination the antibody titers were similar in all vaccinated groups, suggesting that significant interference did not occurred.

Cell-mediated immunity can be also elicited by PCV2 vaccines administered intramuscularly [24, 25]. However, almost no data are available about the development of cell-mediated responses for intradermally administered PCV2 vaccines [26–28]. The result of the present study indicates that intradermal administration of the vaccine resulted in a strong development of PCV2-specific cell-mediated responses. Interestingly, the frequencies of PCV2-specific IFN-γ secreting cells were not affected when comparing the groups with high and low antibody titres vaccinated on the same day. A minor but significant difference was observed when comparing the high titre animals vaccinated at 21 days with the low titre animals vaccinated at 28 days, suggesting a minor level of interference for the higher antibody levels. However, this did not impact the further development of immunity.

In a recent review by Nautrup et al. [29], examining 13 published papers, the authors concluded that vaccination against PCV2 was efficacious to prevent viraemia and to improve the average daily weight gain of vaccinated animals, even when they had high levels of MDA. However, in that review, the authors did not examine the impact on the development of cell-mediated immune responses neither they include intradermally administered vaccines. The results of the present study suggest that, based on the immunological parameters, the intradermal vaccination is efficient in inducing both humoral and cell-mediated responses and thus the efficacy will be probably similar to that of intramuscularly administered vaccines.

## Conclusions

In the conditions of the present study, the development of immunity after intradermal vaccination with Porcilis® PCV ID was not affected by the presence of high levels of MDA the day of vaccination. These results suggest that vaccination of piglets at 3 weeks of age would not be affected by significant interference with MDA in the offspring of PCV2-vaccinated sows.

## Methods

### Design of the experiment

The design of the experiment included two main groups of animals, one with piglets vaccinated at 21 days of age (group 21), and a second with piglets vaccinated at 28 days of age (group 28). Each group was divided into three subgroups: (1) animals with high levels of MDA (subgroup High), that were the offspring of sows vaccinated pre-farrowing with Porcilis® PCV ID (7 and 3 weeks pre-farrowing), (2) low MDA levels, the offspring of unvaccinated sows (subgroup Low) and (3) unvaccinated controls with high or low MDA (subgroup C). Thus, the final design contained the 6 resulting combinations, designated as High21, High28, Low21, Low28, C-High, and C-Low, respectively with 12 animals each in which the High subgroup was the offspring of vaccinated sows and the Low subgroup was the offspring of non-vaccinated sows.

The low-MDA group vaccinated at 28 days (Low28) was considered representative of a standard vaccination schedule at weaning. The number of animals in the experiment was calculated to detect a delayed or decreased response of at least 15% (antibody levels and/or IFN-γ frequencies), compared to the standard group (80% power, 95% confidence level).

### Animals and vaccination procedures

The source farm for piglets was a 600-sow farrow-to-wean farm, operating on a 1-week/batch basis. The farm was unsuspicious for PRRSV (negative results in the routine monitoring), and from previous monitoring of several batches of piglets from 3 to 25 weeks of age by RT-qPCR, it was known that PCV2 circulation was very low since no animals were positive from weaning to slaughterhouse age. To create a scenario for vaccination of the offspring in the presence of high titers of MDA, half of the sows were vaccinated twice with Porcilis® PCV ID at 7 weeks and 3 weeks before the expected date of farrowing. Vaccination was performed as indicated by the manufacturer. The offspring of those vaccinated sows were expected to have high levels of PCV2-specific MDA. The other half of the sows of the selected batch were kept as the low-antibody population.

### Testing of piglets, selection of animals, and allocation to groups

One hundred and sixty-one piglets from 13 sows (randomly selected within a farrowing batch) were bled at 7 days of age. The group of 13 sows was composed by 6 vaccinated (parities 1, 2, 3, 4 and 8) and 7 non-vaccinated sows (parities 1, 2, 4, 7 and 8). Sera were analyzed by ELISA using the PCV2 ELISA kit (Biocheck®, Reeuwijk, The Netherlands) and the PCV2-AlphaLISA test. The PCV2 AlphaLISA test is an in-house (CDS laboratory, Boxmeer, MSD Animal Health) luminescent immunoassay produced using the AlphaLISA technology [30]. Both tests allowed the titration of antibodies and results were expressed as log_10_ of the calculated titre. Selection was performed based on the AlphaLISA results because it resulted in better discrimination of the animals. Animals selected for the high MDA titre subgroups (High21 and High28 plus controls) were the offspring of the vaccinated sows and were above the median of the antibody titre distribution of the total group of sampled piglets. Those in the low titre group were below the median of the total group and corresponded to the offspring of non-vaccinated sows. Animals were ear tagged and then randomly allocated (random numbers) to the vaccination groups to be performed at 21 or 28 days of age, or to the respective control groups.

### Vaccination, transportation to the experimental facilities, sampling, and follow-up

Animals were vaccinated on the farm according to the schedule (21 or 28 days of age). Vaccination was performed intradermally with Porcilis® PCV ID, according to the manufacturer's instructions using a proprietary device (IDAL device). Animals allocated to the controls did not receive the vaccine. After weaning at 28 days, to minimize the possibility of getting infected by PCV2, animals were transported to the experimental facilities of the *Universitat Autònoma de Barcelona*, where they were allocated in climatized pens. Animals remained there until day 42 post-vaccination (42 dpv). Since the experimental pigs did not receive any treatment, were not challenged, nor suffered any condition that required euthanasia, permission was requested from the health authorities to terminate the experiment by sending the animals back to a commercial farm, where they remained until the end of their productive lives.

Pigs were bled on the day of vaccination (0 dpv), at 28 dpv, and 42 dpv. Blood samples were collected in both siliconized and heparinized tubes. Heparinized blood was used to separate peripheral blood mononuclear cells (PBMC), using a gradient density (Histopaque 1.077, Merck).

### PCR, ELISA and ELISPOT

All collected sera were analysed by PCR, using a previously published protocol [31]. Serum samples were analysed for the presence of anti-PCV2 antibodies, using the PCV2 AphaLISA. Half of the animals in the unvaccinated group were randomly assigned to be controls of the animals vaccinated at 21 days of age, while the other half were considered controls of those vaccinated at 28 days of age.

PBMC were analysed for the evaluation of PCV2-specific interferon-gamma (IFN-γ) responses by ELISPOT, using a recombinant Cap protein as the PCV2 antigen (produced by MSD Animal Health). The conditions of the ELISPOT were as reported before, with minor modifications [32]. The optimal concentration of antigen (1:100, equivalent to 5 µg/ml of the cap protein) was determined in a previous titration experiment, using PBMC obtained from two PCV2 vaccinated sows. Unstimulated PBMC and PHA-stimulated PBMC (10 µg/ml) were used as negative and positive controls, respectively (in triplicates). The frequency of PCV2-specific IFN-γ-SC was calculated as the spot counts in antigen-stimulated wells subtracted of the counts in unstimulated wells. Results were expressed as frequencies of PCV2-specific IFN-γ-SC per 10^6^ PBMC.

### Statistical analysis

Statistical analyses were performed using GraphPad Prism 9.5.1. The Kruskal–Wallis test (with Dunn's multiple comparisons test) was used for the comparison of antibody levels or ELISPOT results. Statistical significance was set to *p* < 0.05.

### Supplementary Information

**Additional file 1: Fig. S1.** Distribution of the anti-PCV2 antibody titres using the PCV2 ELISA (Biocheck®)in the animals sampled for the selection of test individuals. Results are expressed as log 10. The bar indicates the median of the distribution.

**Additional file 2: Fig. S2.** Correlation between the results obtained with the pre-study sera using the PCV2 AlphaLISA and the PCV2 ELISA (Biocheck®). The graph shows the correlation of titres and the results of the linear regression analysis.

## Data Availability

Data are property of MSD Animal Health; they can be available however under reasonable request, but some restrictions might apply.

## Abbreviations

ELISPOT: Enzyme-linked immunospot
ID: Intradermal
IFN-γ-SC: Interferon-gamma secreting cells
MDA: Maternally derived antibodies
PBMC: Peripheral blood mononuclear cells
PCV2: Porcine circovirus 2
PHA: Phytohaemagglutinin
