# Supplementary material for: High levels of maternally derived antibodies do not significantly interfere with the development of humoral and cell-mediated responses to Porcine circovirus 2 after intradermal vaccination
Source: Porcine Health Manag. 2023 Sep 15;9:40. doi: 10.1186/s40813-023-00335-9 (PMC10503209; doi:10.1186/s40813-023-00335-9)
Supplement: Supplementary file 1 — Additional file 1: Fig. S1. Distribution of the anti-PCV2 antibody titres using the PCV2 ELISA (Biocheck®)in the animals sampled for the selection of test individuals. Results are expressed as log 10. The bar indicates the median of the distribution. [file 40813_2023_335_MOESM1_ESM.docx]

**Additional file 1: Fig. S1**. Distribution of the anti-PCV2 antibody titres using the PCV2 ELISA (Biocheck®)in the animals sampled for the selection of test individuals. Results are expressed as log 10. The bar indicates the median of the distribution.
