# Supplementary material for: High levels of maternally derived antibodies do not significantly interfere with the development of humoral and cell-mediated responses to Porcine circovirus 2 after intradermal vaccination
Source: Porcine Health Manag. 2023 Sep 15;9:40. doi: 10.1186/s40813-023-00335-9 (PMC10503209; doi:10.1186/s40813-023-00335-9)
Supplement: Supplementary file 2 — Additional file 2: Fig. S2. Correlation between the results obtained with the pre-study sera using the PCV2 AlphaLISA and the PCV2 ELISA (Biocheck®). The graph shows the correlation of titres and the results of the linear regression analysis. [file 40813_2023_335_MOESM2_ESM.docx]

**Additional file 2: Fig. S2.** Correlation between the results obtained with the pre-study sera using the PCV2 AlphaLISA and the PCV2 ELISA (Biocheck®). The graph shows the correlation of titres and the results of the linear regression analysis.
